# FOXO1 Inhibition and FADD Knockdown Have Opposing Effects on Anticancer Drug-Induced Cytotoxicity and p21 Expression in Osteosarcoma Cells

**DOI:** 10.3390/ijms27020935

**Published:** 2026-01-17

**Authors:** Danielle Walker, Antanay Hall, Alexis Bonwell, Nancy Gordon, Danielle Robinson, Mario G. Hollomon

**Affiliations:** 1 Department of Biology, Texas Southern University, Houston, TX 77004, USA; 2 Division of Pediatrics, The University of Texas MD Anderson Cancer Center, Houston, TX 77054, USA

**Keywords:** FOXO1, FADD, cell cycle, osteosarcoma

## Abstract

Forkhead box class O1 (FOXO1) and fas-associated death domain (FADD) regulate cell death pathways and homeostatic processes such as cell cycle progression and apoptosis. FADD phosphorylation promotes nuclear localization of FOXO1, and FOXO1 regulates FADD expression. Therefore, it is plausible that FOXO1 and FADD have synergistic or antagonistic effects on cell cycle regulation and the response to anticancer drug treatment in cancer cells. In the present study, we report that AS1842856-mediated inhibition of FOXO1 reverses anticancer drug-induced cytotoxicity, while FADD knockdown increases anticancer drug-induced cytotoxicity in osteosarcoma (OS). Reversed anticancer drug-induced cytotoxicity was accompanied by G2/M cell cycle arrest and increased expression of p21. The anticancer function of FOXO1 was further supported by the observation that OS cells that express higher basal levels of FOXO1 had increased sensitivity to camptothecin-induced cytotoxicity. FADD knockdown reversed the FOXO1 inhibition-induced increase in p21 expression. The results presented in this study indicate that FOXO1 has a tumor suppressor function, while FADD has a tumor-promoting function in OS following anticancer drug treatment. The experimental approach used in this investigation also indicates that FADD antagonizes the effect of FOXO1 on p21 expression in OS.

## 1. Introduction

Osteosarcoma (OS) is the most prevalent bone cancer that primarily affects adolescents. While the overall survival rates for OS patients have improved over the past 30 years, further improvement is still needed. To develop novel therapeutic options or improve current therapeutic options, a better overall understanding of OS biology is required. Forkhead box O1 (FOXO1) is a transcription factor that regulates the expression of proteins involved in cell growth, differentiation, metabolism, apoptosis, and redox homeostasis [1]. FOXO1 is expressed in bone, and studies suggest that FOXO1 has a significant role in bone homeostasis and health [2]. For example, overexpression of FOXO1 has been reported to increase differentiation, proliferation, and migration of osteoblasts [3]. In addition, Teixeira et al. demonstrated that FOXO1 is a regulator of mesenchymal cell differentiation into osteoblasts [4]. Considering these critical functions in bone biology, it is plausible that FOXO1 has a major function in OS cell cycle regulation and the response to anticancer drug treatment.

Fas-associated death domain (FADD) is an adaptor protein that was first identified for its role in cell death, specifically apoptosis. FADD is also involved in other cell death pathways such as necroptosis [5] and autophagic cell death [6]. FADD has since been linked to non-death cellular processes such as cell proliferation [7], cell cycle regulation [8], cell differentiation [9], and protection against anticancer drug treatment [10]. Cellular localization is considered the determining factor for FADD-induced cell death or cell survival, with cytoplasmic localization associated with cell death and nuclear localization associated with cell survival [11]. p21 is a cyclin-dependent kinase inhibitor (CDKI) that inhibits all cyclin-dependent kinases (CDKs) [12]. FOXO1 regulates the expression of p21 [13], and functional loss of p21 has been implicated in cancer [14]. FADD has been reported to regulate the cell cycle by modulating NFκB-mediated regulation of cell cycle cyclins [15].

Several studies have reported links between FOXO1 and FADD. For example, FADD is reported as a target gene of FOXO1 [16], and FADD phosphorylation promotes FOXO1 nuclear translocation [17]. Considering the role of FOXO1 and FADD in cell death and cell cycle regulation and the reported associations between FOXO1 and FADD, it is plausible that FOXO1 and FADD have a linked effect on OS biology. The synergistic or antagonistic effect of FOXO1 and FADD on OS biology or the response of OS to anticancer drug treatment has not been explored.

The present study set out to investigate the effect of FOXO1 inhibition on cell cycle regulation and the response to anticancer drug treatment in OS. The present study also investigated the combined effect of FOXO1 inhibition and FADD knockdown on cell cycle regulation and anticancer drug-induced cytotoxicity. Here, we report that the inhibition of FOXO1 in OS decreases anticancer drug-induced cytotoxicity and induces G2/M cell cycle arrest. We also report that FOXO1 inhibition-mediated reversal of anticancer drug-induced cytotoxicity is cancer type-dependent. In addition, we present data that indicate that FOXO1 and FADD have opposing functions in cell cycle regulation and in the response to anticancer drug treatment in OS.

## 2. Results

### 2.1. OS Cells Express Different Basal Levels of FOXO1

**Figure 1:**
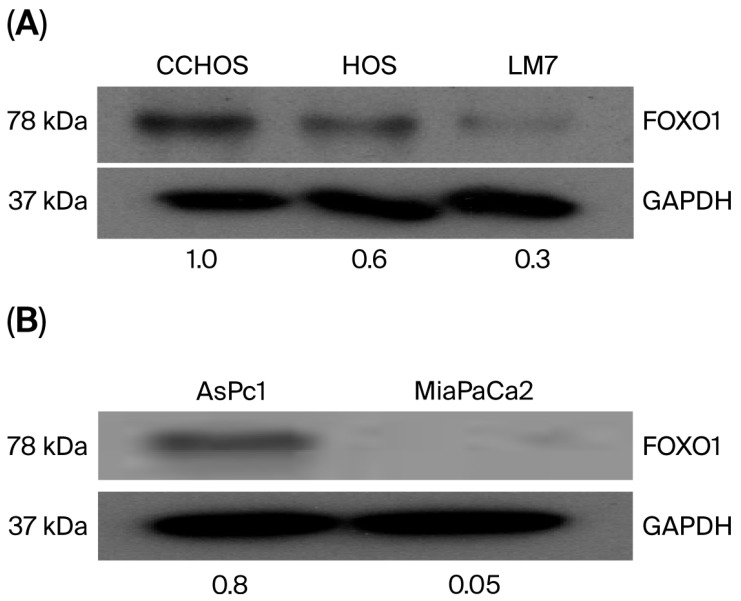
Osteosarcoma (OS) and pancreatic cancer cells express different basal levels of FOXO1 protein. Cells were grown to approximately 70% confluency. Cells were then collected, lysed, and 15 μg of protein was probed for FOXO1 protein expression. FOXO1 basal protein levels: (**A**) OS cells and (**B**) pancreatic cancer cells. GAPDH served as a protein loading control. The immunoblot is representative of immunoblots from two independent experiments.

To begin the present study, basal levels of FOXO1 were determined. Western blot analysis revealed different basal levels of FOXO1 protein in OS. CCHOSD and HOS cells expressed high basal levels of FOXO1 protein (Figure 1A). Pancreatic cancer cell lines, AsPc1 and MiaPaCa2, also expressed different basal levels of FOXO1 protein. AsPc1 cells expressed high basal levels of FOXO1 protein, while MiaPaCa2 expressed very low FOXO1 protein (Figure 1B).

### 2.2. FOXO1 Inhibition Induces Selective Cell Death and Increases p21 Expression

**Figure 2:**
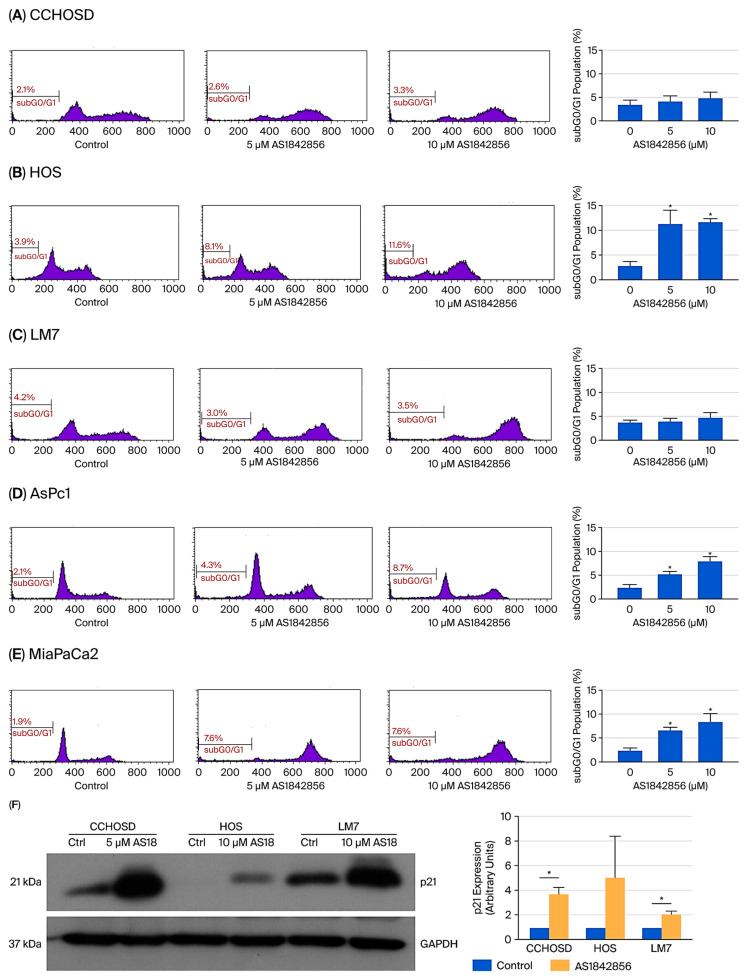
FOXO1 inhibition induces similar overall cytotoxicity in OS cells and pancreatic cancer cells and increases p21 expression in OS cells. Cells were treated with AS1842856 at concentrations indicated in the figures. CCHOSD and HOS cells were treated with AS1842856 for 24 h. LM7, AsPc1, and MiaPaCa2 cells were treated with AS1842856 for 48 h. Following AS1842856 treatment, cells were collected and processed for cell cycle analysis to determine the subG0/G1 cell population. Results from one representative histogram are shown in the left panels. Mean results are shown in the right panels. (**A**) CCHOSD, (**B**) HOS, (**C**) LM7, (**D**) AsPc1, (**E**) MiaPaCa2. Data represent the results of at least three independent experiments, ±SEM. *, *p* < 0.05 was considered significant. (**F**) OS cells were treated with AS1842856 (CCHOSD 5 µM, 24 h; HOS 10 µM, 24 h; LM7 10 µM, 48 h). Cells were then collected, lysed, and 30 μg of protein was probed for p21 expression. The expression of the treatment group p21/GAPDH ratio was determined by densitometry for each cell line and compared to the control group p21/GAPDH ratio, which was normalized to the arbitrary value of one. GAPDH served as a protein loading control. The immunoblot is representative of immunoblots from two independent experiments. Mean results are shown in the right panel. *, *p* < 0.05 was considered significant.

AS1842856 is a specific inhibitor of FOXO1 that inhibits transcriptional activity by binding to the active, non-phosphorylated form of FOXO1 [18]. AS1842856 treatment induced similar overall cytotoxicity in OS cancer cells and pancreatic cancer cells (Figure 2A–E).

### 2.3. Camptothecin Induces Significant Cell Death and Increases p21 Expression

**Figure 3:**
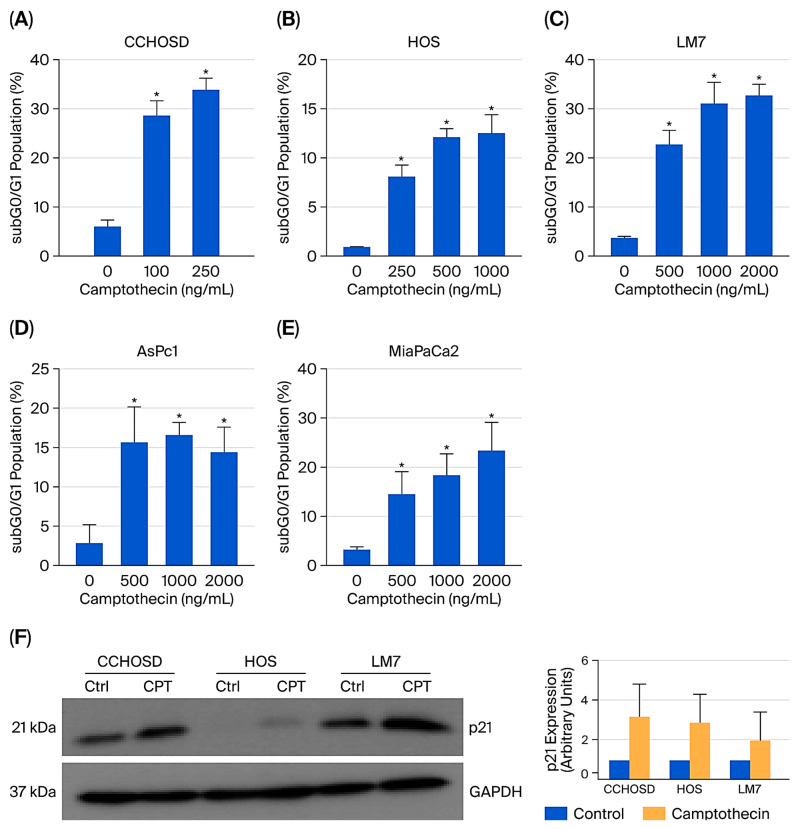
Camptothecin induces cytotoxicity and increases p21 expression. Cells were treated with CPT at concentrations indicated in the figures. CCHOSD and HOS cells were treated with CPT for 24 h. LM7, AsPc1, and MiaPaCa2 cells were treated with CPT for 48 h. Following CPT treatment, cells were collected and processed for cell cycle analysis to determine the subG0/G1 cell population of cells. (**A**) CCHOSD, (**B**) HOS, (**C**) LM7, (**D**) AsPc1, (**E**) MiaPaCa2. Data represent the results of at least three independent experiments, ±SEM. *, *p* < 0.05 was considered significant. (**F**) CPT treatment increases p21 expression. OS cells were treated as follows: CCHOSD, 100 ng/mL CPT (24 h); HOS, 500 ng/mL CPT (24 h); LM7, 2000 ng/mL (48 h). Cells were then collected, lysed, and 30 μg of protein was probed for p21 expression. The expression of the treatment group p21/GAPDH ratio was determined by densitometry for each cell line and compared to the control group p21/GAPDH ratio, which was normalized to the arbitrary value of one. GAPDH served as a protein loading control. The immunoblot is representative of immunoblots from two independent experiments. Mean results are shown in the right panel.

Camptothecin (CPT) is a topoisomerase I inhibitor that binds to topoisomerase I and prevents DNA religation during DNA replication, leading to DNA strand breaks and subsequent cell death [19]. CPT treatment induced significant cytotoxicity in all cell lines investigated (Figure 3).

### 2.4. FOXO1 Inhibition Reverses CPT-Induced Cytotoxicity, and FADD Knockdown Increases CPT-Induced Cytotoxicity

**Figure 4:**
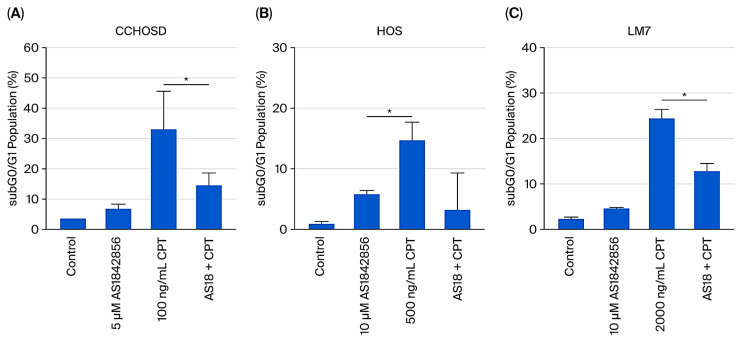
FOXO1 inhibition reverses CPT-induced cell cytotoxicity in OS cells. Cells were treated with either AS1842856, CPT, or a combination treatment of AS1842856 and CPT. Combination treatment included pretreatment with AS1842856 for 1 h followed by treatment with CPT. CCHOSD and HOS cells were treated with AS1842856 or CPT for 24 h. LM7 cells were treated with AS1842856 or CPT for 48 h. Following drug treatment, cells were collected and processed for cell cycle analysis to determine the subG0/G1 cell population of cells. (**A**) CCHOSD, (**B**) HOS, (**C**) LM7. Data represent the results of at least three independent experiments, ±SEM. *, *p* < 0.05 was considered significant.

**Figure 5:**
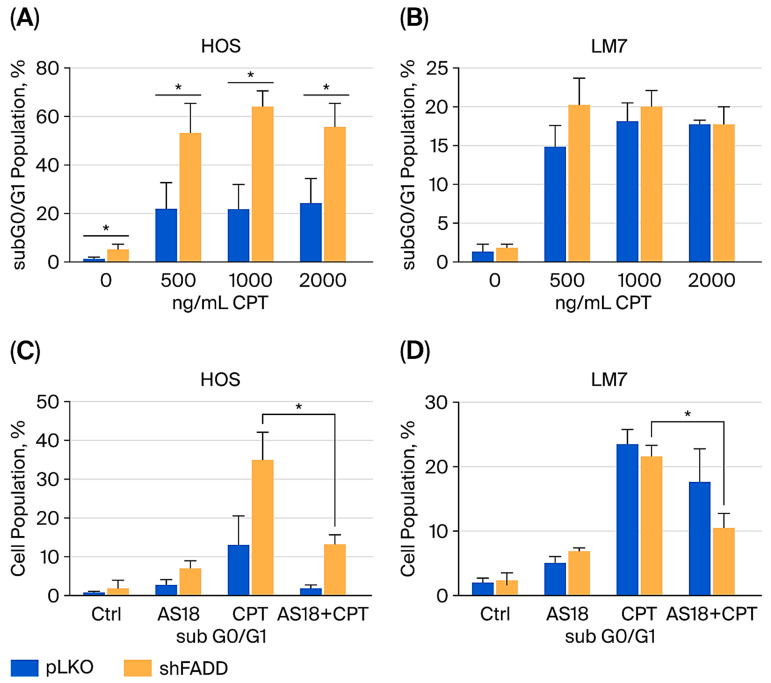
FADD knockdown increases CPT-induced cytotoxicity, which is reversed by FOXO1 knockdown. Wild-type (pLKO) or FADD knockdown (shFADD) cells were treated with CPT. Following drug treatment, cells were collected and processed for cell cycle analysis to determine the cell population in the subG0/G1 phase. (**A**) HOS, (**B**) LM7. pLKO or shFADD cells were pretreated with AS1842856 for 1 h, followed by CPT treatment. Following drug treatment, cells were collected and processed for cell cycle analysis to determine the cell population in the subG0/G1phase. (**C**) HOS, (**D**) LM7. HOS and LM7 cells were pretreated with 10 µM AS1842856. HOS cells were treated with 500 ng/mL CPT for 24 h. LM7 cells were treated with 2000 ng/mL CPT for 48 h. Data represent the results of at least three independent experiments, ±SEM. *, *p* < 0.05 was considered significant.

To investigate the effect of FOXO1 inhibition on anticancer drug-induced cytotoxicity, OS cells were pretreated with AS1842856, followed by treatment with CPT. FOXO1 inhibition reversed CPT-induced cytotoxicity in all OS cell lines investigated (Figure 4). Conversely, FADD knockdown significantly increased OS sensitivity to CPT-induced cytotoxicity in HOS cells (Figure 5A). These results suggest that FADD can have a protective role in OS following CPT treatment, while FOXO1 has a tumor suppressor role following CPT treatment in OS. FOXO1 inhibition reduced the level of CPT-induced cytotoxicity observed in FADD knockdown HOS and LM7 cells (Figure 5C,D).

### 2.5. FOXO1 Inhibition Does Not Reverse CPT-Induced Cytotoxicity in Pancreatic Cancer Cells

**Figure 6:**
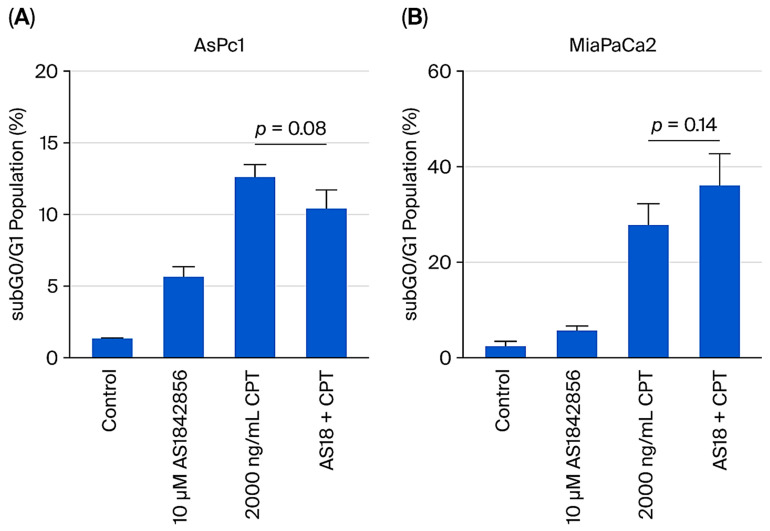
FOXO1 inhibition does not reverse CPT-induced cytotoxicity in pancreatic cancer cells. Cells were treated with either AS1842856, CPT, or a combination treatment of AS1842856 and CPT for 48 h. Combination treatment included pretreatment with AS1842856 for 1 h followed by treatment with CPT for 48 h. Following drug treatment, cells were collected and processed for cell cycle analysis to determine the subG0/G1 cell population of cells. (**A**) AsPc1, (**B**) MiaPaCa2. Data represent the results of at least three independent experiments, ±SEM.

To determine if the FOXO1 inhibition-mediated reversal of CPT-induced cytotoxicity was restricted to OS, two pancreatic cancer cell lines were pretreated with AS1842856, followed by CPT treatment. FOXO1 inhibition did not significantly reverse CPT-induced cytotoxicity in AsPc1 or MiaPaCa2 pancreatic cancer cells (Figure 6). This observation suggests that the effect of FOXO1 inhibition on anticancer drug-induced cytotoxicity is cancer type-dependent.

### 2.6. FOXO1 Inhibition Induces G2/M Cell Cycle Arrest

**Figure 7:**
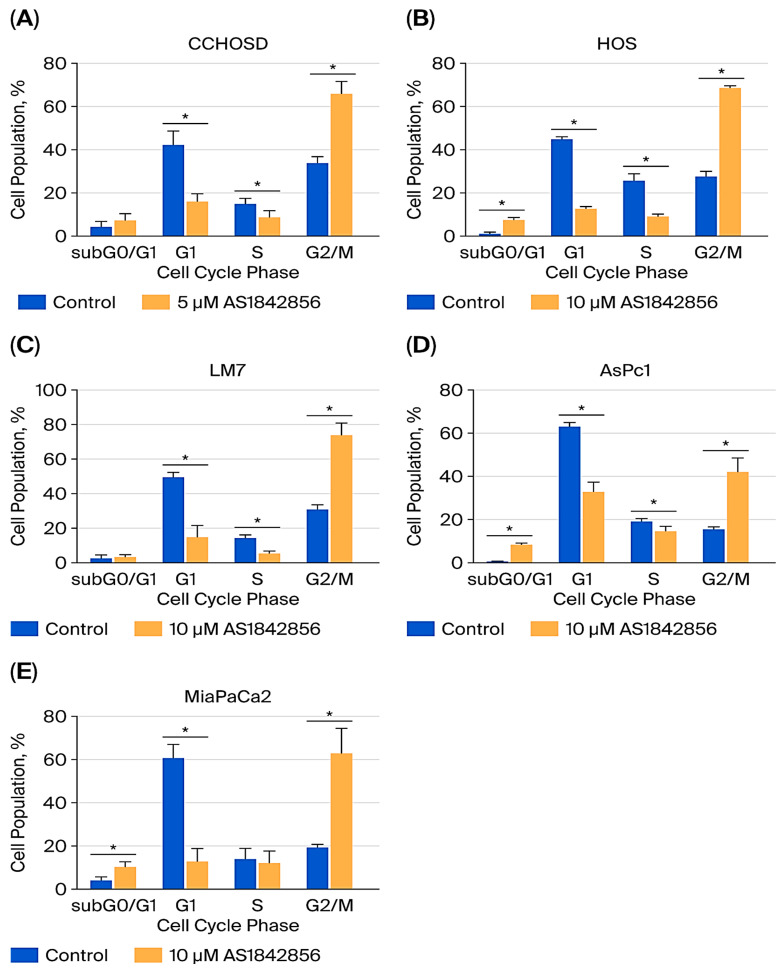
FOXO1 inhibition induces G2/M cell cycle arrest. CCHOSD and HOS cells were treated with AS1842856 for 24 h. LM7, AsPc1, and MiaPaCa2 cells were treated with AS1842856 for 48 h. Following AS1842856 treatment, cells were collected and processed for cell cycle analysis to determine the cell population in each cell cycle phase. (**A**) CCHOSD, (**B**) HOS, (**C**) LM7, (**D**) AsPc1, (**E**) MiaPaCa2. Data represent the results of at least three independent experiments, ±SEM. *, *p* < 0.05 was considered significant.

To explore a possible link between FOXO1 inhibition-mediated reversal of CPT-induced cytotoxicity and cell cycle status, the effect of FOXO1 inhibition on cell cycle progression was investigated. FOXO1 inhibition induced G2/M cell cycle arrest in all OS and pancreatic cancer cell lines investigated (Figure 7).

### 2.7. FADD Knockdown Alters p21 Expression and Reduces FOXO1 Inhibition-Induced p21 Expression

**Figure 8:**
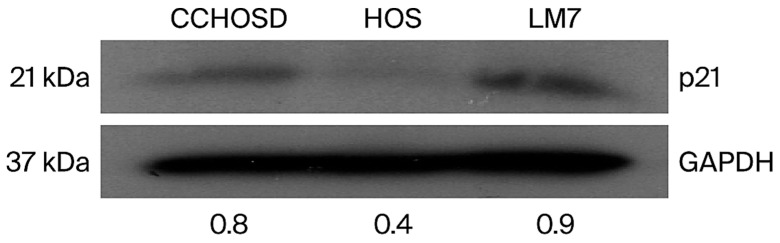
Osteosarcoma cells express different basal levels of cell cycle inhibitors. Cells were grown to approximately 70% confluency. Cells were then collected, lysed, and 30 μg of protein was probed for p21 protein expression. GAPDH served as a protein loading control. The immunoblot is representative of immunoblots from two independent experiments.

**Figure 9:**
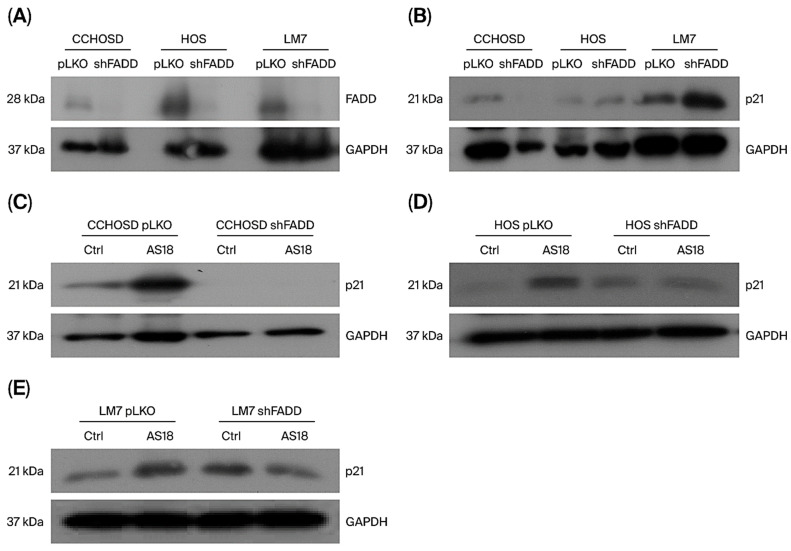
FADD knockdown reduces FOXO1 inhibition-induced p21 expression. Osteosarcoma wild-type (pLKO) or FADD knockdown (shFADD) cells were treated with AS1842856. Cells were then collected, lysed, and 30 μg of protein was probed for p21 protein expression. CCHOSD and HOS cells were treated with AS1842856 for 24 hrs. LM7 cells were treated with AS1842856 for 48 h. (**A**) Lentiviral-mediated knockdown of FADD. (**B**) FADD knockdown alters p21 expression in OS cells. FADD knockdown reduces FOXO1 inhibition-induced p21 expression. (**C**) CCHOSD, (**D**) HOS, (**E**) LM7. GAPDH served as a protein loading control. The immunoblot is representative of immunoblots from two independent experiments.

To investigate the molecular mechanism for FOXO1 inhibition-mediated reversal of CPT-induced cytotoxicity and cell cycle arrest, basal protein expression of p21 was determined. CCHOSD and LM7 cells express higher basal levels of p21 compared to HOS (Figure 8). Western blot revealed a cell line-dependent effect of FADD knockdown on p21 expression in OS (Figure 9B). FADD knockdown reduced p21 expression in untreated CCHOSD cells and increased p21 expression in untreated LM7 cells (Figure 9B). The effect of combined FOXO1 inhibition and FADD knockdown on p21 expression was also determined. FADD knockdown reversed the FOXO1 inhibition-induced increase in p21 expression in CCHOSD and HOS OS cells (Figure 9C–E). These results suggest opposing roles of FOXO1 and FADD in regulating p21 expression in OS.

## 3. Discussion

Several studies have reported a correlation between cancer cell resistance to anticancer drug treatment and FOXO1 expression levels. For example, increased FOXO1 levels are associated with ovarian cancer cell resistance to paclitaxel [20], while ovarian cancer with down-regulated FOXO1 exhibits resistance to cisplatin [21]. These observations underscore the need to better understand the role of FOXO1 in the sensitivity or resistance of cancer cells to anticancer drug treatment. In the present study, Western blot analysis revealed that the OS cell lines studied express different basal levels of FOXO1 (Figure 1). Basal levels of FOXO1 showed a correlation between FOXO1 protein expression and sensitivity to CPT-induced cytotoxicity. Specifically, higher protein expression of FOXO1 correlated with increased sensitivity to CPT-induced cytotoxicity, as indicated by the CPT doses used to induce cytotoxicity (Figure 1 and Figure 3). This correlation was not observed in the two pancreatic cancer cell lines studied. Specifically, the basal level of FOXO1 was higher in AsPc1 cells compared to MiaPaCa2 cells; however, AsPc1 and MiaPaCa2 cells exhibited similar sensitivity to CPT (Figure 1 and Figure 3). These results suggest that FOXO1 inhibition does not affect sensitivity to CPT treatment in pancreatic cancer.

To investigate the effect that FOXO1 inhibition has on the response of OS to anticancer drug treatment, OS cells were pretreated with AS1842856, followed by CPT treatment. FOXO1 inhibition reversed CPT-induced cytotoxicity in all OS cell lines investigated (Figure 4), indicating that FOXO1 contributes to cell death following CPT treatment. Inhibition of FOXO1 also increased p21 expression and induced cell cycle arrest in all OS cells studied (Figure 2 and Figure 7). CPT induces cell death by causing DNA damage during DNA replication; therefore, it is plausible that the reversal of CPT-induced cytotoxicity mediated by FOXO1 inhibition was due to reduced cell cycle progression or cell cycle arrest facilitated by increased p21 expression [22].

The association between FOXO1 and FADD has been previously reported [16,17]. However, the understanding of this interplay on cell cycle regulation or response to anticancer drug treatment has not been investigated in OS. Before investigating the combined effect of FOXO1 inhibition and FADD knockdown on CPT-induced cytotoxicity, the effect of FADD knockdown alone on CPT-induced cytotoxicity was investigated. FADD knockdown increased OS sensitivity to CPT-induced cytotoxicity, suggesting that FADD serves a protective role following CPT treatment (Figure 5A). FADD status has been reported to have opposing effects on the response of cancer cells to anticancer drug treatment. For example, FADD knockdown sensitizes pancreatic cancer to Adriamycin-induced cell death [10]. Conversely, FADD inhibition prevents cisplatin-induced cell death in HT29 and HTCT116 cells and prevents etoposide-induced apoptosis in U937 [23]. In addition, Jurkat cells with FADD knockout are resistant to doxorubicin- or etoposide-induced apoptosis [24]. A comprehensive study that investigates the effect that FADD inhibition has on the response of different cancers to treatment with different anticancer drug classes will contribute to selecting the best anticancer drugs to use in combination therapy that includes FADD inhibition. FOXO1 inhibition significantly reduced the level of FADD knockdown-induced cytotoxicity (Figure 5C,D). This result indicates that FADD knockdown does not alter the tumor suppressor effect of FOXO1.

FOXO1 regulates the cell cycle in multiple cancers by regulating the expression of p16, p21, or p27 [25,26]. To investigate the molecular mechanism responsible for FOXO1 inhibition-induced cell cycle arrest, the basal level of p21 was determined, and a difference in the expression of p21 among the OS cell lines investigated was revealed. p21 is induced by DNA-damaging agents and is a major CDKI that regulates the cell cycle in cancer cells [27,28]. Indeed, CPT treatment increased p21 expression in all OS cell lines investigated (Figure 3F). FOXO1 is well documented to be a positive regulator of p21 expression [25]. Therefore, it was expected that FOXO1 inhibition would decrease p21 expression. However, in the present study, FOXO1 inhibition increased p21 expression in all OS cells (Figure 2). It is plausible that the FOXO1 inhibition-induced increase in p21 was responsible for the observed cell cycle arrest and reversal of CPT-induced cytotoxicity. The observed FOXO1 inhibition-induced increase in p21 expression was reversed by FADD knockdown (Figure 9C–E). The observation that FADD knockdown reversed FOXO1 inhibition-induced increase in p21 expression suggests that FOXO1 and FADD have opposing effects on p21 expression.

## 4. Materials and Methods

### 4.1. Cell Lines, Cell Culture, and Reagents

HOS is a non-metastatic human OS cell line. CCHOSD is a metastatic cell line established from a human osteosarcoma tumor. LM7 is the highly metastatic subline of the low metastatic SaOS2 cell line. AsPc1 and MiaPaCa2 are metastatic pancreatic cancer cell lines. Osteosarcoma and pancreatic cancer cell lines were obtained from the MD Anderson Cancer Center departments of pediatric research and molecular and cellular oncology, respectively. Cells were cultured in DMEM containing 10% FBS and supplemented with antibiotics, non-essential amino acid solution, and MEM vitamin mixture, and they were cultured in an incubator maintained at 5% CO_2_ and 37 °C. Cells were treated with AS1842856 to inhibit FOXO1 transcriptional activity. Lentiviral shRNA targeted against FADD RNA was used to knock down FADD protein expression in the OS cells. Lentivirus containing an empty shRNA vector served as the control for FADD knockdown OS cells. A detailed description of lentivirus generation was provided previously [29]. Cells were cultured in a CO_2_ incubator maintained at 37 °C and 5% humidity. Cells were treated with the drug as indicated in the figures and figure legends. AS1842856 was purchased from Millipore Sigma (Burlington, MA, USA). Camptothecin (CPT) was purchased from ChemWerth (Woodbridge, CN, USA). p21 antibody (2947) was purchased from Cell Signaling Technology (Danvers, MA, USA). GAPDH antibody (sc-32233) and FADD (sc-5559) were purchased from Santa Cruz Biotechnology (Dallas, TX, USA). Mouse secondary antibody (61–6520) and rabbit secondary antibody (31460) were purchased from Millipore Sigma (Burlington, MA, USA). Fetal bovine serum (FBS) and Dulbecco’s Modified Eagle Medium (DMEM) were purchased from VWR International (Radnor, PA, USA). Cell culture supplements were purchased from Invitrogen (Carlsbad, CA, USA).

### 4.2. Cytotoxicity and Cell Cycle Analysis

Cytotoxicity was determined by assessment of the cell population in the sub-G0/G1 area of the cell cycle, which is considered non-viable [30]. Propidium iodide (PI) is a dye that binds to double-stranded DNA by intercalating between base pairs. Cells with degraded DNA (non-viable) have insufficient PI binding and appear in the subG0/G1 population of the cell cycle histogram. Propidium iodide binding to DNA also allows for assessment of cell cycle status [30]. Cells in G0/G1 have DNA that is 2N and have less PI staining than cells in the S stage that are between 2N and 4N. Cells in the G2/M stage have completed DNA synthesis and are 4N; thus, they have the greatest PI staining and the farthest shift to the right in the histogram. A dose-response curve for each drug was carried out to determine the drug treatment dose in downstream experiments (Figure 2 and Figure 3). The middle dose from the dose–response curve was used in downstream experiments for fast-growing cells (CCHOSD and HOS). The highest dose from the dose–response curve was used in downstream experiments for slow-growing cells (LM7, AsPc1, MiaPaCa2). This determination was made due to the mechanism of action for CPT-induced cell death. The AS1842856 dose that induced the greatest cell cycle arrest, but not exceeding 15% cytotoxicity, was used in downstream experiments. 5 µM AS1842856 was used for CCHOSD cells. 10 µM AS1842856 was used for all other cell lines. Following drug treatment, supernatant and cells were collected and centrifuged at 500× *g* for 5 min at 4 °C. The resultant pellet was fixed with cold 70% ethanol and incubated for 24 h at −20 °C. Following fixation, the cells were washed with PBS and centrifuged to remove ethanol and then incubated with PI for 24 h, followed by analysis on a flow cytometer. The Becton Dickinson FACSCalibur flow cytometer (Franklin Lakes, NJ, USA) connected to CellQuest Pro software (version 6.1) was used for flow cytometry experiments and analysis.

### 4.3. Western Blot Analysis

Following drug treatment, supernatant and cells were collected and centrifuged at 500× *g* for 5 min at 4 °C. The resultant pellet was lysed with RIPA lysis buffer containing protease and phosphatase inhibitor cocktail and centrifuged at 14,000× *g* for 15 min at 4 °C. Supernatants were then collected, and total protein was determined by BioRad reagent (BioRad Laboratories, Hercules, CA, USA). The total protein amount listed in figures was resolved in SDS-polyacrylamide gels (SDS-PAGE) and transferred onto nitrocellulose membranes (BioRad Laboratories, Hercules, CA, USA). Membranes were blocked with 5% nonfat milk and then incubated with primary antibody (1:1000 dilution). Membranes were next washed and incubated with the appropriate secondary antibody conjugated to HRP. Following secondary antibody incubation (1:2000 dilution), membranes were washed, and signal was detected with ECL detection reagent (Santa Cruz Biotechnology, Inc., Dallas, TX, USA). GAPDH served as a protein loading control. ImageJ software (version 1.54d) was used for densitometry measurement of Western blot bands.

### 4.4. Statistical Analysis

Results are presented as means ± standard error of the mean (SEM). Experimental data were analyzed using a 2-tailed Student *t*-test in GraphPad Prism statistical software (version 10.2.1). *p*-values less than 0.05 were considered statistically significant.

## 5. Conclusions

The results of this study indicate that FOXO1 has a pro-death function in OS treated with CPT. The results also indicate that the FOXO1 inhibition-induced reversal of CPT-induced cytotoxicity is cancer type-dependent. These conclusions are based on the observation that inhibition of FOXO1 significantly reversed CPT-induced cytotoxicity in all OS cell lines investigated, but not in the pancreatic cancer cell lines. The results also suggest that FADD counters the effect of FOXO1 on p21 protein expression in OS. This conclusion is based on the observation that FADD knockdown reduced the FOXO1 inhibition-induced increase in p21 expression. To the best of our knowledge, this is the first report of AS1842856-mediated inhibition of FOXO1 reversing CPT-induced cytotoxicity in OS. This is also the first report of FOXO1 inhibition or FADD knockdown altering the expression of p21 in OS. The results of this study underscore the need to further investigate the individual and combined roles of FOXO1 and FADD in cell cycle regulation and cancer cell resistance or sensitivity to anticancer drug treatment.

## Data Availability

The original contributions presented in this study are included in the article. Further inquiries can be directed to the corresponding author.

## Abbreviations

The following abbreviations are used in this manuscript:

FOXO	forkhead box-O
FADD	fas-associated death domain
CDK	cyclin-dependent kinase
CDKI	cyclin-dependent kinase inhibitor
CPT	camptothecin
